# Variable frequencies of peripheral T-lymphocyte subsets in the diabetes spectrum from type 1 diabetes through latent autoimmune diabetes in adults (LADA) to type 2 diabetes

**DOI:** 10.3389/fimmu.2022.974864

**Published:** 2022-08-24

**Authors:** Tingting Tan, Yufei Xiang, Chao Deng, Chuqing Cao, Zhihui Ren, Gan Huang, Zhiguang Zhou

**Affiliations:** National Clinical Research Center for Metabolic Diseases, Key Laboratory of Diabetes Immunology (Central South University), Ministry of Education and Department of Metabolism and Endocrinology, The Second Xiangya Hospital of Central South University, Changsha, China

**Keywords:** T-lymphocytes, Th1 cells, CD4^+^ Tcm cells, LADA, T1D, T2D

## Abstract

T lymphocytes are key players in the pathogenesis of autoimmune diabetes. We recruited subjects with T1D (n=81), LADA (n=82), T2D (n=95) and NGT (n=218) and analyzed the percentages of T-lymphocyte subsets, including T helper 1 (Th1), T helper 2 (Th2), T helper 17 (Th17), T cytotoxic 1 (Tc1), regulatory T cells (Tregs), effector T (Teff), naïve T, central memory T (Tcm), and effector memory T (Tem) cells by flow cytometry. LADA patients possessed similar frequencies of IFN-γ^+^CD4^+^ T (Th1), IFN-γ^+^CD8^+^ T and CD4^+^ Teff cells compared with T1D patients, but much lower than those of NGT subjects. Like T2D patients, LADA patients had increased frequencies of CD4^+^ Tem and CD8^+^ Tem cells with respect to T1D and NGT subjects. In LADA patients, Th2 cells were decreased while CD4^+^ Tcm cells were increased compared with NGT subjects. Notably, we observed significant negative correlations between the CD4^+^ Tcm cell frequency and C-peptide in LADA subjects. These data demonstrates that LADA patients possess T-cell subset changes resembling both T1D and T2D and represent the middle of the diabetes spectrum between T1D and T2D. Based on these T-cell subset alterations, we speculate that autoimmunity-induced β-cell destruction and inflammation-induced insulin resistance might both be involved in the pathogenesis of LADA.

## Introduction

Type 1 diabetes (T1D) is a T-cell-mediated autoimmune disease characterized by β-cell destruction and absolute insulin deficiency (1). Latent autoimmune diabetes in adults (LADA) was first introduced in the early 1990s to define a subgroup of patients with adult-onset autoimmune diabetes, with mild and slow progression of β-cell destruction compared to T1D (2, 3). The current classification system subdivides patients mainly according to clinical features such as age at onset, presence of ketosis, and need for insulin replacement, which is equivocal and imprecise. Notably, up to 15% of newly diagnosed T1D patients are negative for islet autoantibodies (4). Similarly, a proportion of adult phenotypic type 2 diabetes (T2D) patients who are autoantibody negative demonstrated autoimmune T-cell responses (5, 6). Thus, it is interesting to investigate the autoimmune etiological differences between different types of diabetes by measuring T cells.

An abundance of CD4^+^ T cells and CD8^+^ cytotoxic T cells was observed in pancreas samples from T1D patients during insulitis (7). Typically, Th1 cells produce interleukin (IL)-2, interferon (IFN)-γ and tumor necrosis factor (TNF)-α and are associated with the progression of T1D development (8). In nonobese diabetic (NOD) mice, Transfer of type 1 T helper (Th1) cells induces T1D (9), whereas blocking IFN-γ prevents T1D (10). Moreover, anti–CD3 monoclonal antibodies have shown clinical efficacy in T1D patients by suppressing Th1 cells (11). Type 2 T helper (Th2) cells produce IL-4, IL-5 and IL-10 and are protective against T1D, since the expression of IL-4 ameliorates diabetes in NOD mice (12, 13). IL-17-secreting T helper 17 (Th17) cells are involved in inflammation and autoimmune diseases, including T1D (14, 15). Transfer of Th17 cells exacerbated insulitis, while neutralization of IL-17 prevented T1D development in animal models (16). Th17 cells also play a crucial role in T2D, possibly by promoting the production of proinflammatory cytokines such as TNF-α to induce insulin resistance in mice models (17). Characterized by their potent regulatory capacity in modulating immunity (18), T regulatory cells (Tregs) have been recognized as protective in both T1D and T2D pathogenesis. In NOD mice, infusion of Tregs reverses diabetes (19), while depletion of Tregs accelerates T1D progression (20). In diet-induced obese (DIO) mice, Tregs might improve obesity-induced insulin resistance (21, 22).

Being predominant in islets with insulitis from NOD mice (23–25) and indispensable for adoptive transfer of the disease (25), CD8^+^ T cells is also a crucial player in T1D. Comprising the majority of CD8^+^ T cells, IFN-γ^+^CD8^+^ T (Tc1) cells are well known for their capacity to secrete IFN-γ, perforin, and granzyme. The pathogenicity of Tc1 cell in T1D has been demonstrated by its cytotoxic activity towards β-cell in animal models both *in vivo* an *in vitro (* 26, 27).

According to the linear differentiation model, the T-cell population could be divided into four categories, namely, naïve, central memory (Tcm), effector memory (Tem) and effector T (Teff) cells. Theoretically, activated, but not naïve, Teff cells drive diabetes pathogenesis (28). Selective depletion of Teff cells prolonged disease remission and preserved islet function in NOD mice (29). Moreover, memory T cells could be a reserve force to damage β cells in the relapse of autoimmunity. T1D patients receiving islet or pancreas allografts years after disease onset still have an association with autoimmunity recurrence, indicating a possible role for long-lasting autoimmunity of memory T cells in diabetes (30).

Therefore, we hypothesize that peripheral T-cell subset frequencies are altered in diabetic patients and might be related to the destruction of β cells. Several studies have reported changes in T cells in diabetic patients (31, 32). Nevertheless, those studies did not comprehensively investigate LADA subjects or compare the whole spectrum of diabetes, from T1D and LADA to T2D, with heathy controls with normal glucose tolerance (NGT). Thus, in this exploratory study with a large sample size, we investigated the frequencies of different T-lymphocyte subsets in patients with T1D, LADA, and T2D compared with subjects with NGT. Furthermore, we made comparisons between patients with different disease durations as well as correlations between T-cell subsets and clinical parameters.

## Materials and methods

### Participants

A total of 258 patients with T1D (n=81, mean disease duration 3.1 ± 3.5 years), LADA (n=82, mean disease duration 6.1 ± 5.9 years), or T2D (n=95, mean disease duration 3.0 ± 3.7 years) and 218 control subjects with NGT were enrolled in our study. All of the participants enrolled in this study were recruited from the Second Xiangya Hospital of Central South University (Changsha, Hunan, China). All patients with diabetes were diagnosed according to World Health Organization (WHO) criteria (33). The diagnostic criteria of T1D were 1) acute-onset ketosis or ketoacidosis with insulin dependency immediately at onset; 2) positivity for at least one islet-specific autoantibody (glutamic acid decarboxylase antibody [GADA], insulinoma-associated protein-2 antibody [IA-2A], or zinc transporter 8 antibody [ZnT8A]); and 3) deficient insulin secretion due to β-cell destruction (1). The diagnostic criteria of LADA (3) were 1) the presence of any of the pancreatic autoantibodies (GADA, IA-2A, and ZnT8A); 2) age ≥30 years at diagnosis; and 3) lack of an absolute requirement of insulin for at least 6 months after diagnosis. The diagnosis of T2D was based on the diagnosis of diabetes according to WHO criteria in the context of insulin resistance and relative insulin deficiency, negative islet autoantibodies and no requirement for insulin treatment immediately at diagnosis. Healthy controls all underwent a standardized 75-g oral glucose tolerance test (OGTT-75 g), and they were enrolled according to the results: subjects with fasting blood glucose levels of <6.0 mmol/L and 2-hour blood glucose <7.8 mmol/L were enrolled as healthy controls with NGT (33, 34). The exclusion criteria were as follows: acute or chronic infection, trauma, surgery or other stress-related events; abnormal renal function with serum creatinine ≥1.5 mg/dL for men and ≥1.4 mg/dL for women; abnormal liver function with elevated serum alanine aminotransferase (ALT) and aspartate aminotransferase (AST) levels (>2-fold the upper limit of normal); autoimmune disease; malignant disease; family history of diabetes mellitus or other autoimmune disease; pregnancy; and immunomodulatory or immunosuppressive treatment (including steroid therapy). The ethics committee of the Second Xiangya Hospital of Central South University approved our study, and all participants gave written informed consent before entering the study.

After physical examination, anthropometric data, including body height (BH), body weight (BW), blood pressure (BP), waist circumference (WC) and hip circumference (HC), were recorded. Fasting venous blood was obtained from all participants and used to assess complete blood counts, liver function, renal function, serum lipid profiles, hemoglobin A_1C_ (HbA_1C_) and C-peptide. Five milliliters of peripheral blood was drawn from each individual for autoantibody and flow cytometry assays.

### C-peptide and HbA1c assays

HbA1c levels were measured by automated liquid chromatography (VARIANT II Hemoglobin Testing System; Bio-Rad Laboratories, Hercules, CA). Fasting C-peptide (FCP) concentrations were determined by chemiluminescence (ADVIA Centaur; Siemens, Munich, Germany).

### Islet autoantibody assays

GADA, IA-2A, and ZnT8A titers were measured by radioligand binding assays as previously described (35). Determined as the 99^th^ percentile of 405 healthy individuals, the cutoff indices of positivity for GADA, IA-2A, and ZnT8A were 18 U/mL, 3.3 U/mL, and 0.011 (ZnT8A index), respectively. Positive samples were tested twice independently for confirmation. According to the islet Autoantibody Standardization Program (IASP2012), the sensitivity and specificity of our GADA, IA-2A and ZnT8 assays were as follows: 78% and 96.7% for GADA, 74% and 96.7% for IA-2A, and 70% and 98.9% for Znt8, respectively.

### PBMC separation and cell surface staining

Venous blood (15 ml) was taken from each subject after an 8–12-hour fasting period, and 5 ml of blood was prepared for peripheral blood mononuclear cell (PBMC) separation within 2 hours. PBMCs were isolated by Ficoll gradient centrifugation, and single-cell suspensions were prepared and then split into 3 cytometry tubes for naïve T/central memory T (Tcm)/effector T (Teff)/effector memory T (Tem), Treg and Th1/2/17/Tc1 cell staining. PBMCs for naïve/memory T and Treg cell staining were stained with two different panels of surface immunostaining antibodies (**Supplemental Table S1**) with optimal dilutions at 4°C in the dark for 20 minutes. Cells were then washed twice with PBS and kept in 4% paraformaldehyde for analysis.

### *In vitro* stimulation and intracellular staining

For Th1/2/17/Tc1 cell staining, isolated PBMCs were first cultured in RPMI-1640 medium supplemented with 50 ng/ml phorbol myristate acetate (PMA), 500 ng/ml ionomycin, 0.67 µl/ml monesin and 10% fetal bovine serum (FBS) at 37°C for 6 hours. Activated cells were then harvested and collected in cytometry tubes. As previously described, PBMCs were stained with cell surface antibodies and subsequently fixed with Fixation Buffer and permeabilized with Intracellular Staining Permeabilization Wash Buffer per the manufacturer’s protocol. Permeabilized cells were then stained with intracellular antibodies (**Supplemental Table S1**) at room temperature in the dark for 40 minutes. Cells were washed twice with Intracellular Staining Perm Wash Buffer and resuspended in PBS for analysis.

For Treg cell intracellular staining, cell surface staining was performed as previously described. Subsequently, Alexa Fluor-anti-FOXP3 antibody was added and incubated at room temperature in the dark for 40 minutes after fixation and permeabilization with a FOXP3 Fix/Perm Buffer Set (BioLegend) per the manufacturer’s protocol. Cells were then washed twice with PBS and resuspended in PBS for analysis.

**Figure 1:**
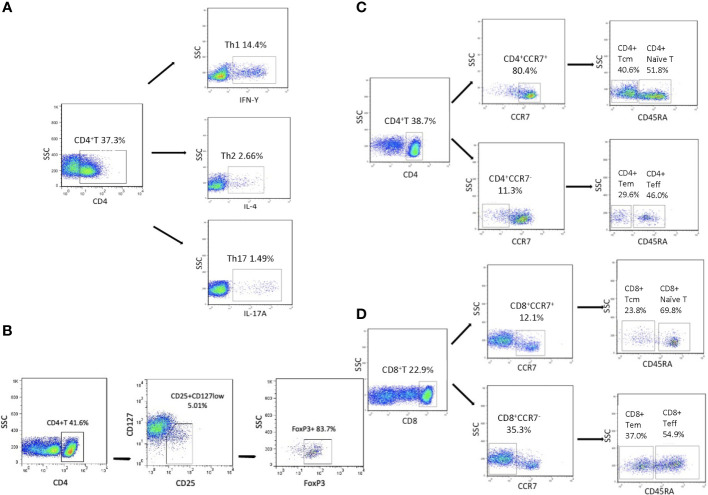
Flow cytometry of peripheral T-cell subsets. **(A)** Representative dot plot showing the gating strategy for IFN-γ^+^CD4^+^ T, Th2 and Th17 cells. **(B)** Representative dot plot showing the gating strategy for Tregs. **(C, D)** Representative dot plot showing the gating strategy for naïve and memory T cells gated on CD4^+^ T/CD8^+^ T cells.

Afterward, flow cytometry analysis was performed in a FACS Canto II analyzer with FACS Diva software (Becton Dickinson, San Jose, CA, USA). The FACS Canto II analyzer was calibrated daily with single fluorochrome-stained samples. At least 30,000 events were collected from each sample in an FSC-A/SSC-A lymphocyte gate and analyzed by FlowJo software Version 7.6 (TreeStar, Inc., Ashlan, OR). By examining the forward- and side-light scatter properties, dead cells were excluded from the analysis. Doublets were excluded by FSC-A/FSC-H properties. Gating strategies for all T-cell subsets examined are described in **Figure 1**. Antibodies used to identify T-cell subsets are as follows (**Supplemental Table S1**): CD4^+^IFN-γ^+^ (Th1 cells), CD4^+^IL-4^+^ (Th2 cells), CD4^+^IL-17A^+^ (Th17 cells), CD8^+^IFN-γ^+^ (Tc1 cells), CD4^+^CD25^+^CD127^low^FOXP3^+^ (Tregs), CD45^+^CD45RA^+^CCR7^+^ (naïve T cells), CD45^+^CD45RA^-^CCR7^+^ (Tcm cells), CD45^+^CD45RA^+^CCR7^-^ (Teff cells), and CD45^+^CD45RA^-^CCR7^-^ (Tem cells).

### Statistical analysis

Data are expressed as the arithmetic means ± standard deviations or as medians (25th-75th percentiles). When data were normally distributed, one-way analysis of variance (ANOVA) was performed and Bonferroni correction was made for multiple testing. The Kruskal–Wallis test was used for nonparametric analysis and Bonferroni correction was made for multiple testing. In addition, covariance analysis was performed to adjust for age and sex. All the significant differences between groups are the results of adjusted analyses unless stated otherwise. Correlations between the T-lymphocyte subset frequencies and other parameters were determined by Spearman nonparametric correlation analysis. Values of p<0.05 were assumed to be statistically significant. Statistical analyses were performed with SPSS 20.0 software (SPSS, Chicago, IL) and GraphPad Prism 5 software (GraphPad Software, San Diego, CA, USA).

## Results

### Anthropometric and metabolic data

The anthropometric data and biochemical parameters of all the groups are summarized in **Table 1**. The age of the NGT subjects was younger than that of the T2D and LADA groups but significantly older than that of the T1D group. The percentage of males in the LADA group was significantly higher than that in the NGT group. T1D patients had the lowest body mass index (BMI) and WC, while T2D patients had the highest BMI and WC among the groups. In addition, T2D patients had higher triglyceride (TG), total cholesterol (TC) and low-density lipoprotein cholesterol (LDL-c) levels than those of the other groups. Fasting blood glucose (FBG) levels were similar in the 3 subgroups of patients with diabetes. The HbA1c concentration was increased in T1D patients with respect to T2D patients. FCP levels significantly declined in the order of T2D, NGT, LADA and T1D.

**Table 1:** Anthropometric and metabolic data of NGT subjects and T2D, LADA and T1D patients.

	NGT (n=218)	T2D (n=95)	LADA (n=82)	T1D (n=81)
Age	41.37 ± 13.52	48.78 ± 12.58***	52.48 ± 14.21***	28.53 ± 16.21***†††‡‡‡
Male (%)	40.4 (88/218)	47.4 (45/95)	59.8 (49/82)**	54.3 (44/81)
BMI (kg/m^2^)	23.10 ± 3.77	24.20 ± 3.39*	22.40 ± 3.15††	19.52 ± 3.17***†††‡‡‡
Waist circumference (cm)	80.67 ± 11.38	87.62 ± 9.97***	82.05 ± 9.67††	73.15 ± 10.15***‡‡‡
Triglyceride (mmol/L) §	1.05 (0.69-1.61)	2.06 (1.21-2.93)***	1.22 (0.80-1.96)†††	0.92 (0.65-1.47)†††
Cholesterol (mmol/L)	4.90 ± 1.00	5.26 ± 1.22*	4.83 ± 1.28†	4.57 ± 1.32†
LDL (mmol/L)	2.74 ± 0.87	3.21 ± 1.06*	2.90 ± 1.10	2.70 ± 1.17
FBG (mmol/L) §	5.09 (4.70-5.40)	7.22 (5.85-9.46)***	8.00 (6.31-10.29)***	8.49 (5.94-12.60)***
HbA_1C_ (%) §	N/A	7.10 (5.90-9.50)	7.40 (6.20-9.73)	8.10 (7.10-11.80)†
HbA_1C_ (mmol/mol) §	N/A	54 (41–80)	57 (44–83)	65 (54–105)†
FCP (nmol/L) §	0.42 (0.33-0.54)	0.53 (0.35-0.76)**	0.31 (0.14-0.50)***††	0.06 (0.03-0.18)***†††‡‡‡

Compared with NGT * p<0.05, **p<0.01, *** p<0.001. Compared with T2D †p<0.05, ††p<0.01, †††p<0.001. Compared with LADA, ‡‡‡p<0.001. §: Data are expressed as the median values (25^th^-75^th^ percentiles) and compared by the Kruskal-Wallis test. Other data are the means ± SDs. LADA, patients with latent autoimmune diabetes in adults; N/A, not applicable; NGT, normal glucose tolerance subjects; T1D, type 1 diabetic subjects; T2D, type 2 diabetic subjects; BMI, body mass index; LDL, low-density lipoprotein cholesterol; FBG, fasting blood glucose; HbA1c, hemoglobin A_1C_; FCP, fasting C-peptide.

### Altered T-lymphocyte subset percentage in subjects with diabetes mellitus

The frequencies of CD8^+^ T, CD8^+^ Teff, Treg and naïve T cells showed no significant differences among the patient groups, regardless of age and sex (**Table 2**, p>0.05).

**Table 2:** T-cell subset frequencies in diabetic patients and NGT subjects.

	NGT (n=218)	T2D (n=95)	LADA (n=82)	T1D (n=81)
CD4^+^ T	40.40 (34.20, 46.60)	43.40 (36.40, 51.50)**	41.00 (32.10, 49.40)	44.20 (38.35, 50.70)
IFN-γ^+^CD4^+^ T	14.8 (10.37, 21.82)	14.2 (9.03, 20.1)	11.7 (9.11, 20.95)*	9.5 9.55 (6.51, 14.75) **
Th2	2.09 (1.34, 2.97)	2.34 (1.39, 3.48)	1.2 (0.73, 2.40) *†	1.65 (1.02, 2.36)
Th17	1.08 (0.75, 1.54)	1.27 (0.82, 2.01) *	1.15 (0.73, 2.17)	0.98 (0.72, 1.44)
IFN-γ^+^CD8^+^ T	42.5 (30.30, 59.00)	39.5 (22.80, 55.07)	41.2 (23.30, 60.80)*	22.7 (13.40, 32.80) ***
Treg	2.67 (1.71, 3.85)	2.97 (2.16, 4.13)	2.81 (1.90, 3.98)	2.62 (1.80, 3.65)
CD4^+^ Naïve T	41.64 (31.41,52.15)	39.41 (23.99,54.98)	34.10 (21.89,47.77)	52.29 (40.50,62.08)
CD4^+^ Tcm	11.50 (7.96,17.04)	14.45 (8.65,21.47)	16.97 (12.26,21.40) **	12.24 (8.91, 18.40)
CD4^+^ Teff	2.76 (1.35, 5.31)	2.34 (1.09, 4.42)	2.21 (0.82, 3.98)*	1.39 (0.76, 2.78)**
CD4^+^ Tem	6.83 (3.33, 10.86)	12.85 (6.89,20.15)***	10.40 (6.55,13.83)**†††	6.11 (2.46, 9.36) †††‡‡
CD8^+^ T	21.70 (17.00, 27.10)	20.60 (16.25, 26.80)	21.50 (15.15, 27.65)	24.00 (15.50, 29.35)
CD8^+^ Naïve T	38.14 (20.39, 51.11)	18.85 (10.43, 35.65)	25.51 (9.42, 47.63)	53.11 (37.04, 66.66)
CD8^+^ Tcm	2.80 (1.92, 3.88)	4.28 (2.18, 6.67)***	3.65 (2.00, 6.05)	1.91 (1.19, 3.29)††
CD8^+^ Teff	7.83 (4.29, 16.07)	13.84 (7.36, 22.66)	7.28 (3.97, 14.42)	5.08 (2.54, 10.60)
CD8^+^ Tem	10.31 (6.04, 15.38)	19.49 (10.04,28.45)***	14.68 (9.22, 23.53) †	8.28 (4.96, 13.24)†††‡

Compared with NGT * p<0.05, **p<0.01,*** p<0.001. Compared with T2D †p<0.05, ††p<0.01, †††p<0.001. Compared with LADA ‡p<0.05, ‡‡p<0.01. Data are expressed as the median values (25^th^-75^th^ percentiles) and compared by the Kruskal-Wallis test. LADA, patients with latent autoimmune diabetes in adults; NGT, normal glucose tolerance subjects; T1D, type 1 diabetic subjects; T2D, type 2 diabetic subjects.

**Figure 2:**
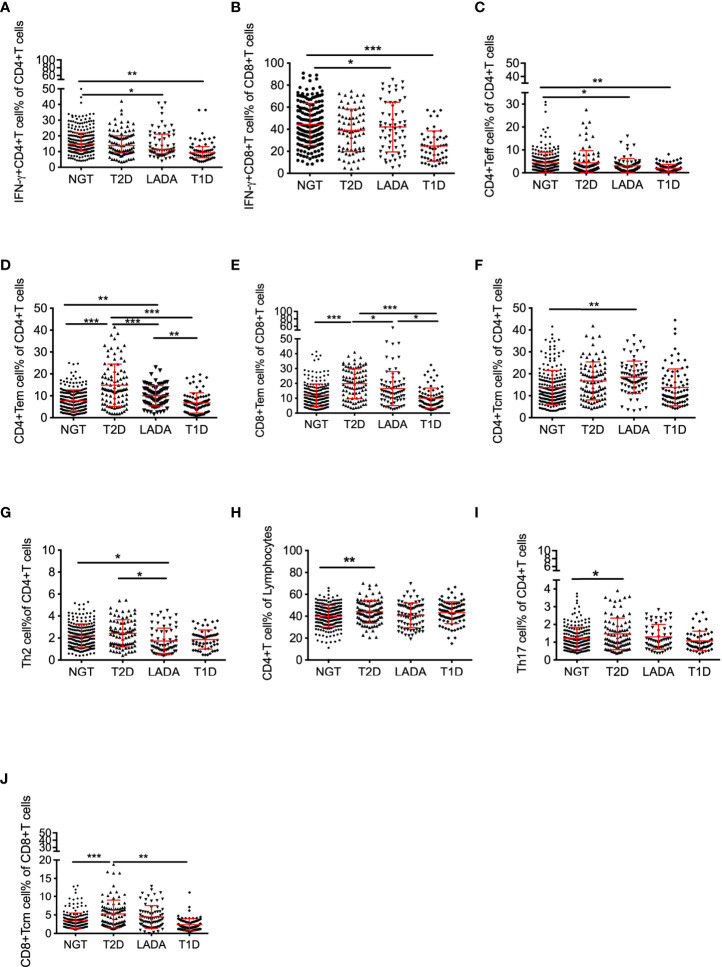
The frequency of peripheral T-cell subsets in diabetic patients and control subjects. The frequency of IFN-γ^+^CD4^+^ T **(A)** and IFN-γ^+^CD8^+^ T **(B)** cells gated on CD4^+^ T or CD8^+^ T cells, CD4^+^ Teff **(C)** and CD4^+^ Tem**(D)** gated on CD4^+^ T cells, CD8^+^ Tem **(E)** gated on CD8^+^ T cells, CD4^+^ Tcm **(F)** and Th2 cells **(G)** gated on CD4^+^ T cells, CD4^+^ T cells**(H)** gated on lymphocytes, Th17 cells **(I)**gated on CD4^+^ T cells, and CD8^+^ Tcm cells **(J)** gated on CD8^+^ T cells in NGT subjects and T2D, LADA and T1D patients as determined by flow cytometry. P values refer to comparisons of data after adjustment for age and sex. Each point represents the percentage of T-cell subsets of an individual. Graphs show the median and interquartile range. *p<0.05, **p<0.01, ***p<0.001.

In LADA patients, the frequencies of IFN-γ^+^CD4^+^ T, IFN-γ^+^CD8^+^ T and CD4^+^ Teff cells were comparable to T1D patients, but significantly decreased with respect to the NGT subjects (IFN-γ^+^CD4^+^ T: T1D vs. NGT, p<0.01; LADA vs. NGT, p<0.05; IFN-γ^+^CD8^+^ T: T1D vs. NGT, p<0.001; LADA vs. NGT, p<0.05; CD4^+^ Teff: T1D vs. NGT, p<0.01, LADA vs. NGT, p<0.05; **Figures 2A-C**).

Being similar to T2D, LADA patients possessed higher frequencies of CD4^+^ Tem and CD8^+^ Tem cells than NGT and T1D subjects (CD4^+^ Tem: T2D vs. NGT, p<0.001; T2D vs. T1D, p<0.001; T2D vs.LADA, p<0.001; LADA vs. NGT, p<0.01; LADA vs. T1D, p<0.01; CD8+ Tem: T2D vs. NGT, p<0.001; T2D vs. T1D, p<0.001; T2D vs. LADA, p<0.05; LADA vs. T1D, p<0.05; **Figures 2D, E**). CD4^+^ Tcm cell frequency was increased in LADA patients compared with that in NGT subjects (LADA vs. NGT, p<0.01, **Figure 2F**). By contrast, the frequency of Th2 cells was decreased significantly in LADA patients with respect to NGT and T2D subjects (LADA vs. NGT, p<0.05; LADA vs. T2D, p<0.05, **Figure 2G**).

In T2D patients, the frequencies of CD4^+^ T, Th17 and CD8^+^ Tcm cells were significantly increased with respect to NGT subjects (NGT vs. T2D:CD4^+^ T cells, p<0.01; Th17 cells, p<0.05; CD8^+^ Tcm cells: NGT vs. T2D, p<0.001; T2D vs. T1D, p<0.01, **Figures 2H-J**).

### Altered T-lymphocyte subset percentage in subjects with new-onset diabetes mellitus

**Figure 3:**
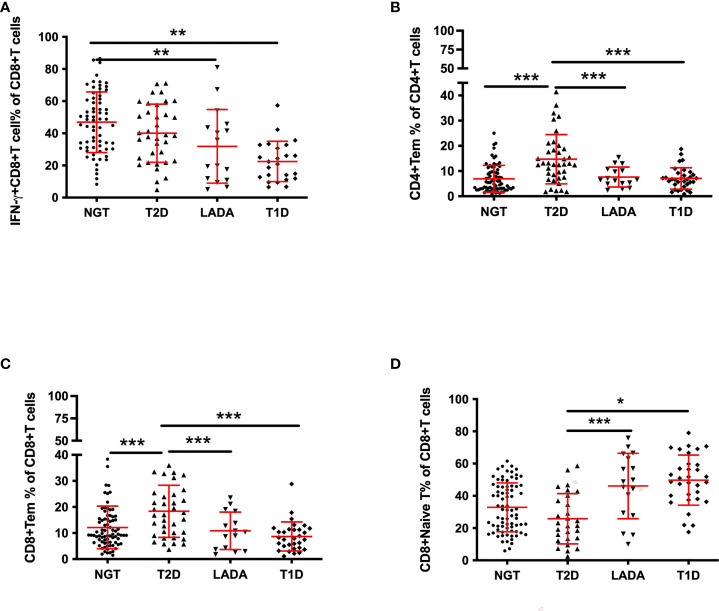
The frequency of peripheral T-cell subsets in new-onset diabetic patients and control subjects. The frequency of IFN-γ^+^CD8^+^ T **(A)** cells gated on CD8^+^ T cells, CD4^+^ Tem cells**(B)** gated on CD4^+^ T cells, CD8^+^ Tem cells**(C)** gated on CD8^+^ T cells, and CD8^+^ naïve T cells **(D)** gated on CD8^+^ T cells in NGT subjects and new-onset T2DM, LADA and T1DM patients as determined by flow cytometry. P values refer to comparisons of data after adjustment for age and sex. Each point represents the percentage of T-cell subsets of an individual. Graphs show the median and interquartile range. *p<0.05, **p<0.01, ***p<0.001.

Furthermore, we analyzed T-cell subsets in patients with disease duration less than one year (new-onset patients). The anthropometric data and biochemical parameters of new-onset patients are presented in **Table 3**. IFN-γ^+^CD8^+^ T cells were less frequent in T1D and LADA patients than in the NGT group (T1D vs. NGT, p<0.01, LADA vs. NGT, p<0.01; **Figure 3A**). The frequencies of CD4^+^ Tem and CD8^+^ Tem cells were increased in T2D compared with the other three groups (CD4^+^ Tem cells: T2D vs. T1D, p<0.001, T2D vs. LADA, p<0.001, T2D vs. NGT, p<0.001; CD8^+^ Tem cells: T2D vs. T1D, p<0.001, T2D vs. LADA, p<0.001, T2D vs. NGT, p<0.001; **Figures 3B, C**). In contrast, CD8^+^ naïve T cells were less frequent in T2D than in the LADA and T1D groups (T2D vs. LADA, p<0.001, T2D vs. T1D, p<0.05; **Figure 3D**) (**Table 4**).

**Table 3:** Anthropometric and metabolic data of new-onset T1DM, LADA, and T2DM patients and NGT subjects.

	NGT (n=80)	T2DM (n=39)	LADA (n=16)	T1DM (n=35)
Age	50.16 ± 12.66	51.27 ± 9.18	45.24 ± 14.23	34.72 ± 15.49*†
Male (%)	48.75 (39/80)	53.84 (21/39)	47.36 (9/19)	48.78 (20/41)
BMI (kg/m^2^)	23.88 ± 3.38	23.81 ± 3.52	21.22 ± 3.14	20.17 ± 3.11*††
Waist circumference (cm)	82.73 ± 10.38	86.25 ± 9.37	78.76 ± 9.67††	76.44 ± 10.15*†
Triglyceride (mmol/L) §	1.13 (0.79, 1.64)	1.60 (1.05, 2.57)*	1.22 (0.89, 1.57)	0.90 (0.77, 1.64)†
Cholesterol (mmol/L)	2.88 ± 0.80	2.95 ± 0.78	2.90 ± 1.10	2.71 ± 1.09
LDL (mmol/L)	2.74 ± 0.87	3.21 ± 1.06*	2.90 ± 1.10	2.70 ± 1.17
FBG (mmol/L)	5.09 ± 0.52	7.59 ± 3.03**	8.75 ± 5.15	9.64 ± 4.46***
HbA_1C_ (%)§	N/A	6.15 (5.75,8.45)	7.4 (5.2,9.72)	8.7 (7.2,12.5)†
HbA_1C_ (mmol/mol) §	N/A	43 (40,69)	57 (33,83)	72 (55,113)†
FCP (nmol/L) §	0.42 (0.34, 0.54)	0.53 (0.38, 0.77)***	0.29 (0.14, 0.49)**	0.16 (0.04, 0.25)**

Compared with NGT * p<0.05, **p<0.01,*** p<0.001. Compared with T2D †p<0.05, ††p<0.01. §: Data are expressed as the median values (25^th^-75^th^ percentiles) and compared by the Kruskal-Wallis test. Other data are the means ± SDs. New-onset: disease duration less than one year. LADA, patients with latent autoimmune diabetes in adults; N/A, not applicable; NGT, normal glucose tolerance subjects; T1D, type 1 diabetic subjects; T2D, type 2 diabetic subjects; BMI, body mass index; LDL, low-density lipoprotein cholesterol; FBG, fasting blood glucose; HbA1c, hemoglobin A_1C_; FCP, fasting C-peptide.

**Table 4:** T-cell subset frequencies in new-onset T1DM, LADA, and T2DM patients and NGT subjects.

	NGT (n=80)	T2DM (n=39)	LADA (n=16)	T1DM (n=35)
CD4^+^ T	41.60 (35.80, 46.90)	44.80 (39.20, 49.90)	46.75 (40.20, 55.60)	42.75 (36.75, 50.50)
IFN-γ^+^CD4^+^ T	15.30 (10.9, 22.00)	16.70 (9.97, 20.80)	11.70 (9.48, 22.00)	10.90 (6.03, 16.50)
Th2	1.94 (1.29, 2.65)	2.16 (2.05, 4.05)	1.62 (0.87, 2.57)	2.23 (1.51, 2.90)
Th17	1.06 (0.67, 1.56)	1.45 (0.94, 2.57)	1.07 (0.78, 1.59)	0.89 (0.59, 1.07)
IFN-γ^+^CD8^+^ T	48.35 (31.2, 61.75)	38.50 (21.90, 50.50)	27.80 (11.15,45.22)**	25.00 (15.35, 31.80)**
Treg	2.52 (1.60, 3.42)	3.06 (2.20, 4.05)	2.75 (1.95, 4.10)	2.40 (1.77, 3.20)
CD4^+^ Naïve T	40.18(31.22, 48.57)	39.41 (27.39, 45.89)	43.13 (24.71, 55.68)	52.27 (39.80, 58.86)
CD4^+^ Tcm	9.38 (6.31, 16.37)	18.63 (8.07, 26.27)	13.14 (7.19,20.85)	12.72 (9.64, 21.10)
CD4^+^ Teff	3.45 (1.38, 6.28)	2.22 (0.80. 5.09)	2.31 (0.77, 4.03)	1.53 (1.04, 3.71)
CD4^+^ Tem	4.91 (2.82, 9.52)	12.99 (9.11,21.07)***	6.48 (4.45, 10.85)†††	6.02 (4.58, 7.99)†††
CD8^+^ T	20.70 (15.40, 24.80)	21.20 (17.90, 26.70)	19.25 (15.97, 27.65)	23.65 (15.75, 29.05)
CD8^+^ Naïve T	31.87 (20.35, 46.83)	22.38 (13.60, 39.44)	48.77 (28.95, 64.55)†††	48.70 (37.73, 58.00)†
CD8^+^ Tcm	3.03 (2.02, 4.11)	4.54 (2.18, 7.07)	4.04 (1.81, 6.62)	1.89 (1.31, 3.97)
CD8^+^ Teff	8.17 (4.65, 16.75)	13.90 (9.50, 21.18)	5.24 (3.42, 8.73)	7.27 (3.76, 13.67)
CD8^+^ Tem	9.21 (6.35, 15.47)	20.46 (12.41,32.82)***	10.00 (3.56,18.56)†††	8.84 (6.15, 11.80)†††

Compared with NGT **p<0.01,*** p<0.001. Compared with T2D †p<0.05, †††p<0.001. Data are expressed as the median values (25^th^-75^th^ percentiles) and compared by the Kruskal-Wallis test. New-onset: disease duration less than one year. LADA, patients with latent autoimmune diabetes in adults; NGT, normal glucose tolerance subjects; T1D, type 1 diabetic subjects; T2D, type 2 diabetic subjects.

### Alteration of T-lymphocyte subsets in autoimmune diabetic patients with different disease durations

To further explore the possible impact of disease duration on T-cell subset frequencies, we divided patients with autoimmune diabetes (T1D and LADA) into 3 subgroups of disease course <1 year, 1-5 years, and >5 years. We compared the frequencies of T-cell subsets among the three subgroups (**Table 5**).

**Table 5:** T-cell subset frequencies in autoimmune diabetic patients with different disease durations.

Disease duration	IFN-γ^+^CD4^+^ T cells (%)	IFN-γ^+^CD8^+^ T (%)	Th2 cells (%)	CD4^+^ Tcm cells (%)
<1 year	10.90 (7.96,17.90)*	25.10 (13.05,35.75)***	1.76 (1.41, 2.88)	12.26 (8.42, 20.08)
1-5 years	10.15 (7.33,13.90)	25.40 (14.70,46.10)	2.04 (1.24, 3.31)	16.04 (11.62, 20.20)
>5 years	10.80 (7.34,18.85)	41.20 (23.60,60.80)	1.01 (0.56, 2.03)**	17.95 (13.34, 24.66)**
NGT	14.80 (10.35,21.85)	42.50 (23.30,59.00)	2.08 (1.33,2.96)	11.54 (7.98, 17.04)

Data are the median values (25^th^-75^th^ percentiles) and were compared by the Kruskal-Wallis test. Compared with NGT * p<0.05, **p<0.01,*** p<0.001.

IFN-γ^+^CD4^+^ T and IFN-γ^+^CD8^+^ T cells were significantly decreased in the <1-year group compared with NGT subjects (Th1: p<0.05; Tc1: p<0.001). In addition, we observed a nonsignificant trend for Tc1 cells to increase with disease duration. Similarly, CD4^+^ Tcm cells tended to increase with disease duration and were significantly increased in the >5-year group compared with the NGT group (p<0.01). Th2 cells were significantly decreased in the >5-year group compared with NGT (p<0.01).

### Correlations between T-lymphocyte phenotype and clinical manifestations

Correlation analysis was conducted to investigate the correlations between T-cell subset frequencies and clinical manifestations. As shown in **Supplemental Table S2**, in patients with diabetes (T1D, LADA, and T2D as a combined group), 1) IFN-γ^+^CD4^+^ T and IFN-γ^+^CD8^+^ T cells were positively correlated with age, 2) Th2 cells were negatively correlated with disease duration, and 3) Th17 cells were positively correlated with blood glucose.

We further examined the correlations between T-cell subsets and clinical parameters in different types of diabetes. Interestingly, in LADA patients, CD4^+^ Tcm cells were positively correlated with FBG (r=0.416, p<0.01), HbA1c (r=0.391, p<0.05) and BMI (r=0.349, p<0.05), while negatively correlated with FCP (r=-0.621, p<0.001) and postprandial C-peptide (PCP) (r=-0.588, p<0.001). CD4^+^ Teff cells exhibited positive correlations with FCP (r=0.468, p<0.01) and PCP (r=0.417, p<0.01) in the LADA group. In T2D patients, CD4^+^ Tem and CD8^+^Tem cells were positively correlated with FBG (r=0.431, p<0.001, for CD4^+^ Tem cells; r=0.266, p<0.05, for CD8^+^ Tem cell) and postprandial blood glucose (PBG) (r=0.315, p<0.05, for CD4^+^ Tem cells; r=0.310, p<0.05, for CD8^+^ Tem cell).

## Discussion

Our research does provide the first comprehensive comparison of different T-cell subsets in the whole diabetes spectrum, comprising subjects with T2D, LADA, T1D and NGT. In our study, we used a large sample size to analyze the majority of T-cell subtypes in subjects with different durations.

The frequencies of IFN-γ^+^CD4^+^ T and IFN-γ^+^CD8^+^ T cells were significantly decreased in LADA and T1D patients compared with NGT patients, while T2D patients had similar frequencies of these cells compared with those of NGT patients. Along with the reduced frequencies of IFN-γ^+^CD8^+^ T cells, there was a trend for an increase in the peripheral IFN-γ^+^CD8^+^ T cell frequency with disease course in autoimmune diabetic patients, although the statistical significance was not obvious. We speculate that the decreased frequencies of IFN-γ^+^CD4^+^ T and IFN-γ^+^CD8^+^ T cells in LADA and T1D patients may be due to the sequestration of these pathogenic cells in pancreatic lymph nodes (PLNs) after disease onset, which is supported by the persistence of diabetogenic T cells PLNs in NOD mice (36). Consistent with the reduction in peripheral IFN-γ^+^CD4^+^ T cells, we observed reduced frequencies of CD4^+^ Teff cells in LADA and T1D patients compared to those of NGT or T2D subjects. Indeed, our analysis of CD4^+^ T-cell subsets showed that IFN-γ^+^ T cells represent the majority of CD4^+^ Teff cells. Published data support a pathogenic role for Teff cells in T1D, and ablation of Teff cells could significantly preserve β-cell function (29). Importantly, T1D patients receiving islet or pancreas allografts still had an association with autoimmunity recurrence years after disease onset, indicating the possibility of long-lasting autoimmunity of pathogenic T cells in autoimmune diabetes (30). Collectively, it is plausible that CD4^+^ Teff cells persistently reside in the PLNs and serve as a reserve force to damage β cells in the relapse of autoimmunity.

LADA and T2D patients possessed much higher frequencies of CD4^+^ Tem cells and CD8^+^ Tem cells with respect to NGT and T1D subjects. Consistent with our finding, other investigators have reported increased memory T cells in obese and T2D patients (37, 38). Indeed, increases in CD4^+^ Tem cells and CD8^+^ Tem cells in visceral adipose tissue have been demonstrated in DIO mice. Speculatively, obesity induces low-grade inflammation and an excessive release of proinflammatory cytokines, activating naïve T cells into Tem cells. More importantly, activated Tem cells might drive the increase in proinflammatory M1 macrophages and exacerbate metainflammation, damaging both β cells and key components in insulin signaling pathways. Thus, obesity-related meta-inflammation may be characterized by an increase in Tem cells in both LADA and T2D patients.

To date, autoantibodies have been used to diagnose autoimmune diabetes in clinical practice, but they do not necessarily indicate insulitis. Instead, the appearance of autoreactive T cell could be the immunologic hallmark of autoimmunity. We observed that like T1D, LADA patients possess immunological features of decreased IFN-γ^+^CD4^+^ T and IFN-γ^+^CD8^+^ T cells; and meanwhile, bearing a resemblance to T2D, LADA patients had chronic inflammation-related changes of increased Tem cells. In line with these shared common features on T-cell subsets frequencies with T1D and T2D, we have previously found that LADA patients shared cytokine profile similarities with both T1D and T2D patients (39). Also, previous studies by us found that LADA patients represented mixed innate immune cell features of T1D and T2D, demonstrated by the gradually increasing neutrophil cell counts from T1D, LADA to T2D (40). Notably, our team reported that LADA has heterogenous autoimmunity in a GADA titer-dependent manner between T1D and T2D. We found that β-cell function decline in subjects with high GADA titer resembled that in T1D patients, while subjects with low GADA titer were similar to T2D patients (41). Collectively, these findings corroborate the view that LADA represents the middle of the diabetes spectrum between T1D and T2D. Autoimmune attack against islet β-cells and insulin resistance caused by chronic systemic inflammation are both involved in the process of LADA.

We found an increased frequency of peripheral Th17 cells specifically in T2D patients, which is in accordance with previous reports (31, 42). Excessive IL-17 production could upregulate the gene expression of inflammatory cytokines, including TNF-α, IL-6 and IL-1β, by activating the NF-κB pathway. These cytokines further promote serine phosphorylation of insulin receptor substrate-1 (IRS-1) to dampen insulin signaling, ultimately inducing insulin resistance (16). Moreover, a reduction in HbA1c values in T2D patients was reported to be associated with a decrease in IL-17 concentration (43). Collectively, these findings suggest that Th17 subset dominates in the progression of insulin resistance and T2D pathogenesis.

We observed decreased frequencies of Th2 cells in LADA patients compared with T2D and NGT subjects. To our knowledge, an altered frequency of Th2 cells in LADA patients has not been reported previously. The decrease in protective Th2 cells might indicate the relationship of this subset of cells to the pathology of LADA. In addition, we found no differences in Treg frequencies among T1D, LADA, T2D and NGT groups. As for Tregs in LADA, other investigators have reported increased expression of FoxP3 in CD4^+^CD25^+^T cells (44), or similar frequencies of CD4^+^CD25^+^CD127^-^ FoxP3^+^Tregs among LADA, T1D, T2D and healthy controls (45).In contrast, in 2006, our team reported decreased FoxP3 mRNA expression in LADA patients (46). These discrepancies require further study including investigation of Treg functions. Notably, LADA patients possess much higher frequencies of CD4^+^ Tcm cells than those of NGT subjects. Furthermore, the CD4^+^ Tcm cell frequency was negatively correlated with islet function solely in LADA patients after adjustment for variables including age, sex and BMI. Our finding is reminiscent of studies of abatacept treatment in T1D patients, which revealed that an increase in CD4^+^ Tcm cells was significantly associated with C-peptide decline (47). Collectively, CD4^+^ Tcm cells could act as a reservoir to elicit the function of CD4^+^ Teff cells to propagate β-cell destruction in LADA. LADA patients are perceived as ideal for immune modulation therapies since their β-cell function decline slower compared to T1D. Thus, based on our finding of the increase of CD4^+^ Tcm cell and its correlation with islet function, we could explore the effect of targeted ablation of autoreactive CD4^+^ Tcm cells on β-cell protection in LADA patients in the future.

However, the results we observed were from the periphery. Further sampling and analysis of T cells in the pancreas and pancreatic lymph nodes are needed. Another limitation of our study is that our data were based on total T cells instead of autoreactive T cells. In this sense, investigation into the frequencies and phenotypes of T cells reactive to β-cell antigens, including insulin, proinsulin, or islet antigen (IA)-2, by HLA tetramers (multimer) staining techniques would be of great value. In addition, it should be noted that there are distinct differences in age among T1D, LADA, T2D and NGT groups and we thus performed covariance analysis to adjust for age. Besides, we have not included CD3 or other dump markers in our flowcytometry panels, whereas addition of such markers may help to define T-cell subset alterations more precisely. Furthermore, a prospective study with long-term follow-up comprising first-degree relatives of diabetic patients and individuals with prediabetes could be useful in further investigating the role of T-cell subsets through the integral disease course of diabetes.

In summary, our large-scale study revealed that LADA patients possess T-cell subset alterations resembling both T1D and T2D. Our findings suggest that β cell loss due to Th1 response disorder and insulin resistance caused by chronic inflammation both participate in the pathoetiology underlying LADA. A thorough examination of T-cell subsets might help the accurate classification approaches of diabetes and provide foundations for T cell subset-directed interventions for LADA in the future.

## Data availability statement

The original contributions presented in the study are included in the article/**Supplementary Material**. Further inquiries can be directed to the corresponding author.

## Ethics statement

The studies involving human participants were reviewed and approved by ethics committee of the Second Xiangya Hospital of Central South University. Written informed consent to participate in this study was provided by the participants’ legal guardian/next of kin.

## Author contributions

TT performed experiments, analyzed data, wrote the manuscript, and contributed to discussion. YX designed the study, researched data, edited the manuscript and provided critical review. CD, ZR, and CC performed the experiments. GH contributed to the islet autoantibodies assay. ZZ designed the study, contributed to discussion, and edited the manuscript. All authors contributed to the article and approved the submitted version.

## Funding

This study was supported by grants from the National Natural Science Foundation of China (No. 82000849, No.81800745) and Natural Science Foundation of Hunan Province (No. 2021JJ40810, No. 2021JJ40826).

## Acknowledgments

The authors thank Dr. Bin Zhao for reading the manuscript. Dr. Bin Zhao: Department of Metabolism and Endocrinology, The Second Xiangya Hospital, Central South University

## Conflict of interest

The authors declare that the research was conducted in the absence of any commercial or financial relationships that could be construed as a potential conflict of interest.

## Publisher’s note

All claims expressed in this article are solely those of the authors and do not necessarily represent those of their affiliated organizations, or those of the publisher, the editors and the reviewers. Any product that may be evaluated in this article, or claim that may be made by its manufacturer, is not guaranteed or endorsed by the publisher.
