# Supplementary material for: Variable frequencies of peripheral T-lymphocyte subsets in the diabetes spectrum from type 1 diabetes through latent autoimmune diabetes in adults (LADA) to type 2 diabetes
Source: Front Immunol. 2022 Aug 24;13:974864. doi: 10.3389/fimmu.2022.974864 (PMC9449581; doi:10.3389/fimmu.2022.974864)
Supplement: Supplementary file 1 [file Table_1.docx]

**Supplemental Table S1 Antibodies used for flow cytometry.**

| Flurochrome and cell markers | | | | | | | Main subpopulations identifiable |
| --- | --- | --- | --- | --- | --- | --- | --- |
| FITC | PE | APC | APC-Cy7 | PerCP-Cy5.5 | PE/Cy7 | Alexa Fluor-488 |  |
| C45RO | CCR7 | CD45RA | CD4 | CD8 | CD45 | / | CD4, CD8-naïve and memory T-cell subsets |
|  | CD25 | CD127 | CD4 | CD8 | CD69 | FOXP3* | Tregs |
| IFN-γ* | IL-17* | IL-4* | CD4 | CD8 | / | / | Th1, Th2, Th17 and Tc1 cells |

***: Antibodies for intracellular staining.**

**Supplemental Table S2 Correlation analysis between peripheral T-cell subset frequencies and anthropometric and metabolic variables and islet autoantibodies for all groups**

|  | Unadjusted | | | | | Adjusted for age, sex, and BMI | | | | |
| --- | --- | --- | --- | --- | --- | --- | --- | --- | --- | --- |
|  | IFN-γ^+^  CD4^+^ T | Th2 | Th17 | IFN-γ^+^  CD8^+^ T | Treg | IFN-γ^+^  CD4^+^ T | Th2 | Th17 | IFN-γ^+^  CD8^+^ T | Treg |
| Age (years) | 0.227*** |  |  | 0.511*** |  | 0.206* |  |  | 0.475*** |  |
| Sex |  |  |  |  |  |  |  |  |  |  |
| Disease duration (years) |  | -0.255*** |  |  |  |  | -0.262*** |  |  |  |
| Body weight (kg) |  |  |  |  |  |  |  |  |  |  |
| BMI (kg/m2) | 0.142** |  |  | 0.168*** |  | / |  |  | / |  |
| sBP (mmHg) |  |  |  |  |  |  |  |  |  |  |
| dBP (mmHg) |  |  |  |  |  |  |  |  |  |  |
| Triglycerides (mmol/L) |  |  |  |  |  |  |  |  |  |  |
| Total cholesterol (mmol/l) |  |  |  | 0.180*** |  |  |  |  | / |  |
|  | Unadjusted | | | | | Adjusted for age, sex, and BMI | | | | |
|  | IFN-γ^+^  CD4^+^ T | Th2 | Th17 | IFN-γ^+^  CD8^+^ T | Treg | IFN-γ^+^  CD4^+^ T | Th2 | Th17 | IFN-γ^+^  CD8^+^ T | Treg |
| HDL cholesterol (mmol/L) |  |  |  | 0.118* |  |  |  |  | / |  |
| LDL cholesterol (mmol/l) |  |  |  |  |  |  |  |  |  |  |
| HbA1c (%) | -0.177* |  |  |  |  | / |  |  |  |  |
| FBG (mmol/L) |  |  | 0.111* |  |  |  |  | 0.128* |  |  |
| PBG (mmol/L) |  |  | 0.102* |  |  |  |  | 0.102* |  |  |
| FCP (nmol/L) | 0.226*** |  |  | 0.299** |  | / |  |  | / |  |
| PCP (nmol/L) | 0.320** |  | 0.213** | 0.389** |  | / |  | / | / |  |
| GADA |  |  |  |  |  |  |  |  |  |  |
| IA-2A |  |  |  |  |  |  |  |  |  |  |
| ZnT8 |  |  |  |  |  |  |  |  |  |  |

Correlation analyses between peripheral T-cell subset frequencies and anthropometric metabolic variables in patients with diabetes (T1D, LADA, and T2D as a combined group) were performed using Spearman test. * p<0.05, **p<0.01,*** p<0.001.
